# Tissue-Specific Whole Transcriptome Sequencing in Castor, Directed at Understanding Triacylglycerol Lipid Biosynthetic Pathways

**DOI:** 10.1371/journal.pone.0030100

**Published:** 2012-02-03

**Authors:** Adrian P. Brown, Johan T. M. Kroon, David Swarbreck, Melanie Febrer, Tony R. Larson, Ian A. Graham, Mario Caccamo, Antoni R. Slabas

**Affiliations:** 1 School of Biological and Biomedical Sciences, Durham University, Durham, United Kingdom; 2 The Genome Analysis Centre, Norwich Research Park, Colney, Norwich, United Kingdom; 3 Department of Biology, Centre for Novel Agricultural Products, University of York, York, United Kingdom; Max Planck Institute for Chemical Ecology, Germany

## Abstract

**Background:**

Storage triacylglycerols in castor bean seeds are enriched in the hydroxylated fatty acid ricinoleate. Extensive tissue-specific RNA-Seq transcriptome and lipid analysis will help identify components important for its biosynthesis.

**Methodology/Findings:**

Storage triacylglycerols (TAGs) in the endosperm of developing castor (*Ricinus communis*) seeds are highly enriched in ricinoleic acid (18:1-OH). We have analysed neutral lipid fractions from other castor tissues using TLC, GLC and mass spectrometry. Cotyledons, like the endosperm, contain high levels of 18:1-OH in TAG. Pollen and male developing flowers accumulate TAG but do not contain 18:1-OH and leaves do not contain TAG or 18:1-OH. Analysis of acyl-CoAs in developing endosperm shows that ricinoleoyl-CoA is not the dominant acyl-CoA, indicating that either metabolic channelling or enzyme substrate selectivity are important in the synthesis of tri-ricinolein in this tissue. RNA-Seq transcriptomic analysis, using Illumina sequencing by synthesis technology, has been performed on mRNA isolated from two stages of developing seeds, germinating seeds, leaf and pollen-producing male flowers in order to identify differences in lipid-metabolic pathways and enzyme isoforms which could be important in the biosynthesis of TAG enriched in 18:1-OH. This study gives comprehensive coverage of gene expression in a variety of different castor tissues. The potential role of differentially expressed genes is discussed against a background of proteins identified in the endoplasmic reticulum, which is the site of TAG biosynthesis, and transgenic studies aimed at increasing the ricinoleic acid content of TAG.

**Conclusions/Significance:**

Several of the genes identified in this tissue-specific whole transcriptome study have been used in transgenic plant research aimed at increasing the level of ricinoleic acid in TAG. New candidate genes have been identified which might further improve the level of ricinoleic acid in transgenic crops.

## Introduction

Plant seeds contain a diverse range of fatty acids which have uses in the food, health and industrial sectors. These fatty acids accumulate in seed triacylglycerols (TAGs) in which each of the three carbons of the glycerol backbone can be linked to a different fatty acid. From an industrial perspective however it is desirable to have TAGs enriched with one specific fatty acid so they can be used directly following hydrolysis, circumventing the requirement to fractionate individual fatty acid species. Ricinoleic acid (12-hydroxyoctadec-*cis*-9-enoic acid; 18:1-OH) is the major fatty acid in *Ricinus communis* (castor bean) seeds where it constitutes up to 90% of the fatty acids found in TAGs. One of the principle uses of 18:1-OH is as a raw material for the production of Nylon-11 (N-11) which is resistant to organic solvents at high temperature. This property makes N-11 a highly desirable polymer for the manufacture of tubing used to carry hydraulic fluids in automotive engines. Over 50,000 tons of 18:1-OH, sourced from castor, is used annually for the production of N-11 but castor has a number of undesirable attributes as an industrial crop. It contains the highly allergenic 2S albumin and the toxin ricin, is restricted climatically to certain growth areas and is a non-determinant plant which is not conducive to mechanical harvesting. An added problem is the inconsistent price of castor oil in the commercial market which can greatly affect the economics of N-11 production. To overcome these problems, research has been directed at understanding the metabolic pathways of tri-ricinolein (TAG with 18:1-OH at all three positions on the glycerol backbone) biosynthesis from oleic acid in developing castor seeds with a view to engineering its synthesis in alternative oilseed crops.

Conversion of oleic acid to 18:1-OH in plant seeds occurs in the endoplasmic reticulum (ER) whilst the fatty acid is attached to the *sn-2* position of phosphatidylcholine (PC) [1]. This reaction is catalysed by oleate 12-hydroxylase (phosphatidylcholine 12-monooxygenase). The gene encoding this enzyme was discovered using an expressed sequence tag (EST) sequencing approach on developing castor beans [2]. Identification was based on the knowledge that the reaction was not catalysed by a cytochrome P450 but probably by a protein related to acyl-lipid desaturases [3] and that 18:1-OH synthesis occurs exclusively in seeds. Subsequent transformation of tobacco and mass spectrometry of fatty acids demonstrated proof of function of the identified sequence. The amount of 18:1-OH produced in tobacco seeds was low, only 0.1%, but subsequent experiments with the castor and related *Lesquerella fendleri* hydroxylases produced seeds with 12.8 and 15.6% hydroxylated fatty acids respectively in *Arabidopsis thaliana* and *Brassica napus* [4].

Two sets of reactions can reduce 18:1-OH levels in both castor and transgenic plant TAGs: (a) competition for the pool of oleic acid by oleate desaturase and (b) further metabolism of 18:1-OH by elongation reactions and additional desaturases to give for example 18:2-OH and 20:1-OH fatty acids. The effect of reducing these competing reactions was studied by expressing oleate 12-hydroxylase in *Arabidopsis* with *fad* (oleate desaturase) and *fae* (fatty acid elongation) mutations [5]. Double mutant lines accumulated 19.2% hydroxylated fatty acids in mature seeds which is substantially lower than the level in castor oil and points to a requirement for additional modification of TAG-synthetic reactions to increase 18:1-OH levels in transgenic plants.

**Figure 1:**
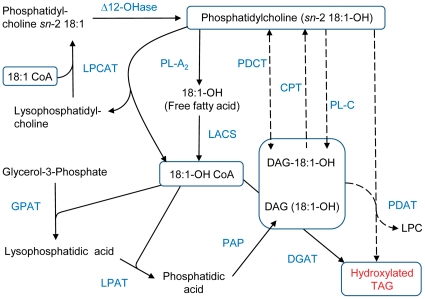
Pathways of triacylglycerol biosynthesis. Reactions in the formation of hydroxylated TAGs via the acyl-CoA dependent (solid arrows) and independent (dashed arrows) pathways are shown. Lipid substrates are abbreviated: 18:1, oleic acid; 18:1-OH, ricinoleic acid; LPC, lysophosphatidylcholine; DAG, diacylglycerol. Enzyme abbreviations are: Δ12-OHase, oleate-12-hydroxylase; LPCAT, 1-acylglycerol-3-phosphocholine acyltransferase; PL-A_2_, phospholipase A_2_; LACS, long chain acyl-CoA synthetase; GPAT, glycerol-3-phosphate acyltransferase; LPAT, lysophosphatidic acid acyltransferase; PAP, phosphatidic acid phosphatase; DGAT, diacylglycerol acyltransferase; CPT, CDP-choline:diacylglycerol cholinephosphotransferase; PL-C, phospholipase C; PDAT, phosphatidylcholine diacylglycerol acyltransferase.

The classical pathway for TAG biosynthesis in seeds is the acyl-CoA dependent Kennedy pathway which involves three membrane-bound acyltransferases and a phosphatidic acid phosphatase [6], [7] (Figure 1). These enzymes are encoded by gene families in plants and each isoenzyme may have a different specificity for both acyl donor and acceptor. Individual acyltransferases can therefore influence incorporation of specific fatty acids into TAG and transfer of them between plant species has been shown to alter seed oil compositions [8], [9]. Two diacylglycerol acyltransferase (DGAT) isoenzymes are present in castor: RcDGAT1 and RcDGAT2. *In vitro* experiments demonstrated that RcDGAT2 can utilise di-ricinolein and 18:1-OH-CoA substrates and its expression profile in developing castor bean is consistent with a specific involvement in tri-ricinolein biosynthesis [10]. This was subsequently demonstrated by studies in which introduction of *RcDGAT2* into *Arabidopsis* lines expressing oleate 12-hydroxylase resulted in elevation of 18:1-OH seed content from 17 to nearly 30% [11].

Identification of enzymes important in the formation of 18:1-OH-CoA substrates for acyl-CoA dependent reactions is complicated by the occurrence of enzyme isoforms and alternative biosynthetic routes. Acyl editing, the exchange of fatty acids between polar membrane lipids such as PC and the acyl-CoA pool without net glycerolipid synthesis [12], [13], [14], can occur by two pathways (Figure 1). Modified fatty acids may be removed from PC by phospholipase A_2_ (PL-A_2_) and the released fatty acids then activated to acyl-CoAs by long-chain acyl-CoA synthetase (LACS). Alternatively, the reverse reaction of acyl-CoA:lysophosphatidylcholine acyltransferase (LPCAT) can generate acyl-CoAs from PC [15].

An acyl-CoA independent TAG-synthetic pathway is active in a number of plant species, including castor [16]. In this, phospholipid:diacylglycerol acyltransferase (PDAT) catalyses transacylation of the *sn-2* fatty acid from PC onto the *sn-3* position of diacylglycerol (DAG), with lysophosphatidylcholine (LPC) as a co-product (Figure 1). A variety of PDAT isoforms exist in both *Arabidopis* and castor and the effects of transferring castor PDATs into *Arabidopsis* co-expressing oleate 12-hydroxylase has been investigated. Transformation with *RcPDAT1* produced up to 27% hydroxylated fatty acids in Arabidopsis seeds [17], [18]. An alternative acyl-CoA independent process for the incorporation of hydroxylated fatty acids into TAG from PC is removal of the phosphocholine head-group to form diacylglycerol (which can be further acylated by PDAT). Three different mechanisms are possible for this step involving either phospholipase C, the reverse action of CDP-choline:diacylglycerol cholinephosphotransferase (CPT) [19] or the recently identified phosphatidylcholine:diacylglycerol cholinephosphotransferase (PDCT/ROD1) [20]. Determining which isoform(s) of these enzymes are expressed in castor seed may be important for engineering of 18:1-OH synthesis. Metabolic labelling experiments have recently shown that in *Arabidopsis thaliana* the main flux of TAG synthesis (between 60 and 93%) is through PC and the majority of newly synthesised fatty acids in soybean embryos are incorporated into glycerolipids via PC acyl editing rather than the Kennedy pathway [13], [21]. In castor the relative importance of acyl-CoA dependent and independent pathways and the proportions of *de novo* synthesised DAG versus PC-derived DAG contributing to TAG synthesis are unknown.

Although the activity of RcDGAT2 and RcPDAT1 go some way to explaining how 18:1-OH accumulates to such a large extent in castor seeds, there is still much to understand. This includes answers to questions such as: does the acyl-CoA pool size in developing seeds reflect the final oil composition (which might suggest little selectivity in TAG-synthetic enzymes); are there specific acyl-CoA pools used for TAG synthesis; is there metabolic channelling of intermediates in TAG-biosynthetic protein complexes and are there seed-specific enzyme isoforms for the synthesis of tri-ricinolein? Comparison of gene expression between developing castor seeds and other tissues, which can make TAG, but do not contain 18:1-OH, may help to pinpoint enzymes involved in a 18:1-OH specific TAG pathway if it exists.

We have previously generated ESTs from a normalised, subtracted castor seed library and obtained 4901 non-redundant sequences (EMBL/Genbank GE632284–GE637184). Normalisation via subtraction allows greater sequencing depth by compensating for highly expressed sequences but comparative information, which could be highly informative in selecting important genes in tissue specific metabolism, is lost. In this whole transcriptome sequencing study we sought to obtain quantitative gene expression data from a variety of castor tissues, selected on the basis of differing lipid metabolism. Developing seeds synthesise storage TAGs while germinating seeds use them as an energy source via β-oxidation and form new cellular membranes. TAG metabolism is limited in leaves but they require extensive photosynthetic membrane lipids. Male developing flowers were selected as they do contain TAGs but do not synthesise 18:1-OH. The castor genome has recently been sequenced [22], providing a reference genome for mapping reads from a next generation sequencing platform. Illumina's sequencing by synthesis technology was chosen for this study as it produces deep coverage of expressed sequences and reliable quantitative data [23]. We anticipate comparison of gene expression will aid in selection of candidate genes for transformation studies to test their potential for increasing 18:1-OH levels in oilseeds.

## Results and Discussion

### 18:1-OH-CoA is not the dominant acyl-CoA in developing seed endosperm

**Figure 2:**
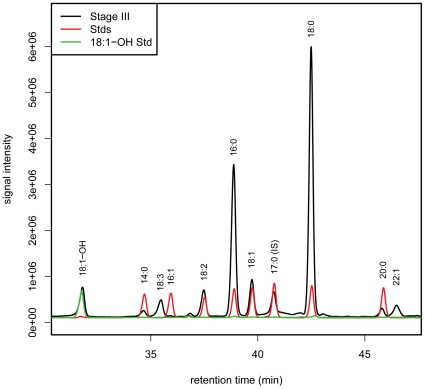
Acyl-CoA analysis of developing castor endosperm. A chromatogram of fluorescent acyl-CoA derivatives from developing castor endosperm stage III is shown - dark trace. Results from analysis of synthesised 18:1-OH-CoA (green line) and other acyl-CoA standards (red trace) are superimposed to confirm the indicated peak identities in the endosperm sample.

Oil enriched in 18:1-OH is synthesised in the endosperm of developing castor seed. The fatty acid content of seed oils may directly reflect the composition of acyl-CoA pools available to TAG biosynthetic enzymes, or factors such as enzyme substrate selectivity and metabolon-formation could channel certain fatty acids into TAG. The acyl-CoA species present in developing castor bean endosperm were analysed using a fluorimetric method developed for plant tissues [24]. The results show that 18:1-OH-CoA is not a dominant species in this tissue (Figure 2). It forms ∼4% of the detected acyl-CoA peak area at seed developmental stage III [25], the earliest obvious stage of endosperm formation, and the maximum value in later stages is 8% (Table 1). 18:1-OH-CoA is made from 18:1-CoA by acyl-editing on PC and the proportions of these species are broadly equivalent during stages of oil deposition (Table 1). During this period the most abundant acyl-CoA species in castor endosperm are 16:0 and 18:0, which is somewhat surprising, given that tri-ricinolein is the major TAG in castor beans. This implies that TAG synthesis must involve selective incorporation of 18:1-OH, possibly by acyl-CoA independent pathways, or the presence of a discrete acyl-CoA pool which is incorporated into TAG.

**Table 1:** Acyl-CoA levels in developing castor endosperm.

Stage^a^	18:1-OH CoA	18:1 CoA	16:0 CoA	18:0 CoA	18:2 CoA
III	4.25 (0.34)	7.18 (0.89)	32.25 (3.04)	31.73 (4.36)	7.52 (0.62)
IV	5.17 (0.84)	6.59 (0.42)	23.74 (1.88)	31.34 (0.35	6.21 (0.64)
V	4.90 (0.22)	7.97 (0.52)	33.05 (3.21)	26.65 (4.44)	4.45 (0.31)
VI	8.01 (0.89)	6.61 (0.71)	28.89 (3.25)	28.08 (1.23)	7.89 (1.71)
VII	3.82 (0.42)	5.17 (0.38)	33.63 (0.40)	31.35 (4.09)	4.68 (0.50)
VIII	7.03 (0.37)	4.23 (0.51)	27.93 (1.49)	26.12 (3.45)	4.13 (0.74)

The percentage of the total acyl-CoA peak area represented by specified acyl-CoAs are shown for castor endosperm stages during seed development. Analysis of fluorescent acyl-CoA derivatives was as in [24] and average values from three extractions and analyses for each stage are shown, together with the standard error in brackets (*n* = 3).

a Endosperm samples were staged according to [25].

### Triacylglycerols are made in both developing seed endosperm and pollen, but the fatty acid composition is different

**Figure 3:**
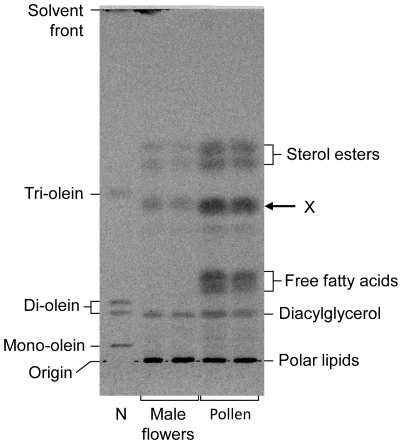
TLC analysis of neutral lipids from castor developing male flowers and pollen. Lipid extracts were applied to a silica TLC plate which was developed with hexane/diethyl ether/acetic acid (70∶30∶1) before iodine-staining. The position of lipid standard components (lane N) and proposed nature of resolved sample lipids are shown. Lipid X and proposed sterol esters were purified for further analysis.

Castor oil consists of 80–90% 18:1-OH and FAME analysis of mature castor beans reveals an equivalent high proportion of this fatty acid (Table 2). Di- and tri-ricinoleoyl TAGs are synthesised in the endosperm and 18:1-OH can be detected in this tissue at an early stage of seed development (stage III, [25]). In an attempt to find an alternative castor tissue that makes TAG, but does not incorporate hydroxylated fatty acids into it, a number of lipid extracts were analysed (Table 2). Cotyledons from developing seeds contain substantial amounts of 18:1-OH and their lipid metabolism is thought to be similar to that of endosperm. Neither developing male flowers nor pollen however contain 18:1-OH and when neutral lipids from these were analysed by TLC a lipid with similar mobility to standard tri-olein was identified (Marked X in Figure 3). This was subsequently purified from pollen by Flash Chromatography (Figure S1) and FAME analysis revealed it contained predominantly 16:0, 18:2 and 18:3 fatty acids (Table 2, Lipid X).

**Table 2:** Fatty acid analysis of castor tissues and purified lipid X.

Fatty acid	Early endo-sperm (4)	Mature bean (2)	Cotyledon (4)	Developing ♂ Flowers (2)	Pollen (2)	Lipid X (2)
16:0	11.8	1.2	2.1	18.7	13.8	22.4
18:0	1.3	1.1	1.3	0.9	0.9	6.1
18:1	16.8	3.8	10.0	6.7	1.5	6.2
*16:3* a	*1.7*			*1.2*	*0.7*	
18:2	32.2	5.0	11.3	32.5	11.7	18.6
18:3	9.0	0.5	0.9	19.8	29.9	36.1
20:1		0.3	1.1	1.4	1.0	
*22:0*				*2.5*	*3.2*	
22:1				1.3	3.9	
*24:0*				*0.9*	*1.3*	
nd^b^				4.3	13.0	
*24:1*				*0.9*	*3.1*	
18:1-OH	26.2	86.4	73.4	-	-	
nd				6.1	7.3	
nd				2.2	2.3	

Average peak-area percentages of fatty acid methyl ester derivatives are listed. The number of separate sample extractions and analyses are listed in brackets, except for lipid X, where the result from two GC injections of the purified fraction is shown.

a Italicised entries are probable fatty acid designations, although the species did not exactly co-chromatograph with standards.

b Indicates the identity of the molecular species is not known but likely wax or hydroxylated fatty acid derivatives from pollen protective layers.

**Figure 4:**
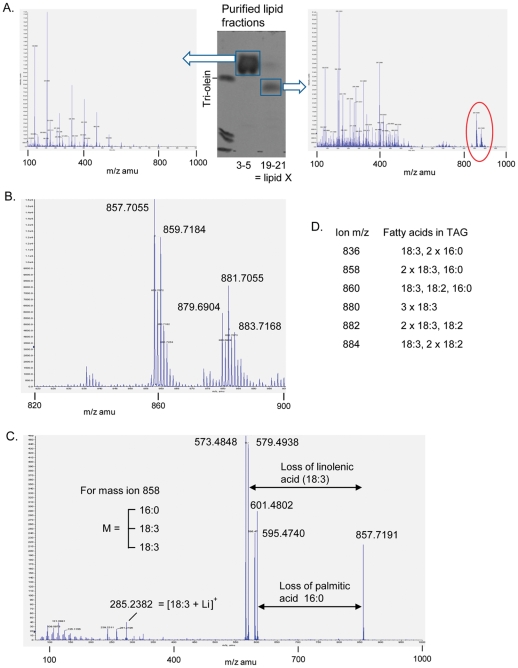
MS analysis of purified neutral lipids from pollen. Purified neutral lipids from pollen were analysed by TLC and used for electrospray MS analysis in the presence of lithium (panel A). Ions between 830 and 900 amu in the lipid X fraction (highlighted in red and Panel B) were selected for fragmentation and the diagnostic fragments produced, such as those in Panel C from the 858 mass ion, allowed determination of fatty acid composition of proposed TAG species (Panel D).

To further investigate the nature of this neutral lipid species and to establish it was indeed TAG, electrospray mass spectrometry analysis in the presence of lithium was carried out [26]. Addition of lithium enhanced formation of ion species at 830–900 amu in purified lipid-X which were not detected in other purified neutral lipids from pollen (Figure 4A, B). Collision associated dissociation of ions in this region led to formation of diagnostic fragments consistent with neutral loss of free fatty acids and fatty acid lithium salts from the parent ions, as previously described for MS analysis of TAGs [26]. An example of such fragmentation is shown, together with the fatty acid composition of proposed TAG species which are consistent with the mass ions observed in this region of the spectrum (Figure 4C, D). We conclude that lipid X is a mixture of TAG species containing saturated and polyunsaturated fatty acids which are different from those found in castor oil. We have thus identified pollen-producing male flowers as a castor tissue which is capable of making TAG without 18:1-OH in it. Comparison of gene expression between this and developing endosperm might lead to the identification of genes and proteins involved in specific pathway(s) of 18:1-OH incorporation into TAG.

### Experimental design and transcriptome analysis

Selection of castor tissues was based on differences in their lipid metabolism, increasing the likelihood of informative results about enzymes important for seed oil synthesis. Two stages of developing seed endosperm, stages II/III and stage V/VI [25] were chosen as representative of early and mid-late stages of oil deposition, the level of lipid-biosynthetic transcripts might be expected to rise in the latter. Leaves synthesise fatty acids for photosynthetic and other membranes but oil does not accumulate in them to an appreciable extent. Some TAG is found in Arabidopsis leaves, but it accounts for less than 1% of lipid [27] and it remains to be determined whether TAG mobilisation plays an important role in this tissue. Germinating seeds were selected as they are degrading rather than synthesising storage lipids and transcriptome information from them might also be of use to those studying pathways of lipid degradation and in particular peroxisome metabolism and biogenesis. Finally, male flowers synthesise non-hydroxylated TAGs which are found in pollen.

Tissue samples from at least five different plants were pooled before RNA extraction to minimize the number of sequencing runs. RNA-Seq analysis was performed with the Illumina GAII sequencing by synthesis platform to obtain deep coverage of transcribed genes and statistically robust comparative data. The availability of a castor genome sequence provided a resource for data processing although it should be noted this was from a non-commercial castor line whereas here we use the commercial variety 99N89I. A summary of read numbers and mapping information from this study is in Table S1. Full data-sets are deposited and available at the European Nucleotide Archive (http://www.ebi.ac.uk/ena), accession number ERA047687.

RNA-Seq reads from the five castor tissues were mapped to release 0.1 of the castor assembly with expression detected for 18713 of the annotated 31221 gene models in the castor genome (see Materials and Methods). Normalised RNA-Seq fragment counts were used to measure the relative abundance of transcripts with expression reported in Fragments Per Kilobase of exon per Million fragments mapped (FPKM). Calculated FPKM expression values and results from a test statistic to determine significant changes in expression between pairs of samples are shown in Table S2. Our primary interest was in potential oil-biosynthetic enzymes in castor seeds and the results discussed below predominantly relate to lipid metabolism. Inspection of the castor genome identified 143 genes coding for enzymes of fatty acid synthesis and acyl (glycerolipid and wax) metabolism and expression data for these are in Table S3. This also includes 25 selected storage proteins, which provide a useful reference for transcriptional activity in developing seeds. A heatmap of FPKM normalised log_2_-transformed transcription values for genes in this table was generated (Figure S2), to aid analysis of lipid metabolic genes with similar expression patterns.

High expression of lipid-related genes in a particular tissue suggested a possible specific role in lipid metabolism and warranted further investigation. Analysis of interesting candidate enzymes included identification of the closest Arabidopsis homologue, in order to gain insight into possible gene function. Although some lipid metabolic genes have been manually annotated [22], designation of most castor gene models is based on automated homology- and domain-searches and more reliable functional information may be present in database entries for Arabidopsis orthologues.

Gene expression between pairs of tissues was assessed using the Cuffdiff algorithm [28] in order to try and identify genes important for tri-ricinolein synthesis and preferably those co-expressed with oleate 12-hydroxylase in castor seed.

### Transcriptome analysis of two developing seed stages demonstrates high expression of lipid biosynthetic genes and enhanced seed storage gene expression at the later stage

Early (free nuclear, stage II/III) and later (cellular, stage V/VI) endosperm samples of developing seeds reveal which lipid metabolic genes are transcribed in a tissue synthesising tri-ricinolein, their changes during seed development and how expression relates to storage protein transcript levels. We have divided selected gene models into functional groups including fatty acid synthase, TAG synthesis and seed storage proteins for analysis (Table S3) and the main findings are discussed below.

#### 1. Transcripts for Type II Fatty Acid Synthase enzymes and oleate-12-hydroxylase are abundant in developing seed

From Table S3, under the heading of fatty acid synthesis, it can be seen that transcription of all the enzymes required for fatty acid biosynthesis from acetyl-CoA is elevated in the endosperm. Read counts for constituents of both acetyl-CoA carboxylase (ACCase) and fatty acid synthase (FAS) are substantially higher than in other tissues. Genes encoding subunits of heteromeric ACCase, (α-CT, β-CT, BC and BCCP) are much more highly expressed than those for the homomeric, multi-functional ACCase. All FAS components have at least one isoform with transcript levels comparable to heteromeric ACCase and enzyme isoforms more highly expressed in castor endosperm are easily identifiable. Thioesterase A terminates fatty acid synthesis at a chain length of 18 carbons and its 5-fold higher FPKM value compared to thioesterase B, which releases C16 fatty acids, reflects demand for C18 fatty acids for seed-oil synthesis. Stearoyl-ACP desaturase completes oleic acid synthesis in the plastid and elevated transcription of several genes encoding it occurs in the endosperm. Transcription levels for all of these enzymes in stage II/II are apparently sufficient to meet demands for fatty acid synthesis in developing castor seeds and read counts generally decrease in the later endosperm stage.

The situation is different for the oleate 12 hydroxylase (Table S3 - Fatty acid modification) where transcription nearly doubles in the later stage. Differential regulation of oleate 12-hydroxylase and the related oleate 12-desaturase genes occurs during seed development; expression of the latter decreases in stage V/VI and there is 6-fold difference in calculated FPKM values at this stage, compared to a 2 fold difference at the earlier stage. Our data for these two genes is in agreement with previous expression profiling in castor seeds [29], [30]. If this transcriptional control is reflected at the protein and enzyme activity level, there would be decreased competition between hydroxylation and desaturation reactions in later stage endosperm. The putative cytochrome P450 (29791.m000529) is highlighted here due to high ratios of FPKM values in endosperm:leaf and endosperm:male flower. The Arabidopsis orthologue of this is a fatty acid (omega1) hydroxylase and while it is likely this enzyme is involved in cutin or suberin synthesis in castor, its substrates and products are unknown at present.

#### 2. Analysis of seed endosperm RNA-Seq data reveals differences in transcript levels for acyl-CoA dependent TAG-biosynthetic genes

Fatty acids synthesised in the plastid are exported and activated to acyl-CoA thioesters by acyl-CoA synthetase (ACS) enzymes, alternatively known as fatty acid CoA ligases. Transcription of seven castor ACS genes was detected and one, 29908.m006186, is clearly the most highly expressed in endosperm, with an FPKM value of 232 in both developmental stages (Table S3 - Acyl-CoA activation). This encodes a homologue of long chain acyl-CoA synthetase 9 (LACS9) from Arabidopsis, which has recently been shown to functionally overlap with the ER-localised LACS1 in seed-oil synthesis [31]. Acyl-CoAs are thought to be bound by acyl-CoA-binding proteins as they move between organelles, such as the plastid and endoplasmic reticulum, and expression of the castor acyl-CoA binding protein encoded by gene 29827.m002594 is highest in the endosperm.

The first steps of the acyl-CoA dependent Kennedy pathway are catalysed by glycerol-3-phosphate acyltransferase (GPAT) and lysophosphatidic acid acyltransferase (LPAT). No clear GPAT candidate with a specific role in seed TAG synthesis emerges from the RNA-Seq data as read counts for predicted GPATs are relatively low in the two developing endosperm stages (Table S3 - Kennedy pathway). *GPAT9* (30122.m000357) is the most likely to be important in castor oil synthesis as it has the highest FPKM value amongst *GPATs* in endosperm, although equivalent expression in other tissues is evident. Read counts for 27810.m000646, the most highly expressed *LPAT* in the endosperm, suggest an involvement in storage oil metabolism but again, it is also transcribed to an appreciable level in other tissues. Somewhat surprisingly gene 29666.m001430, the closest orthologue of *LPAT* genes encoding seed-specific microsomal LPATs found in species that accumulate unusual fatty acids in their seed oil [32], [33], has low transcript levels in castor endosperm.

Phosphatidic acid phosphatase (PAP) converts PA to diacylglycerol (DAG) in the Kennedy pathway. Multiple PAP types and isoforms exist in plants but their exact role in TAG biosynthesis is unclear. No PAP candidate for TAG synthesis was identified from in this study and the castor homologues of Arabidopsis PAH1 and 2, which are expressed strongly in seeds [34], are transcribed in all tissues. The final step in TAG assembly by the Kennnedy pathway is the acylation of DAG by diacylglycerol acyltransferase (DGAT). In castor, single-copy gene models encode two classes of ER-associated DGAT as well as an orthologue of a soluble DGAT in peanut [10], [35], [36]. Expression of membrane-bound RcDGAT2 - 29682.m000581, is substantially higher than the two other *DGAT*s in developing castor seeds (Table S3).

#### 3. Expression of gene isoforms involved in acyl-PC remodelling and acyl-CoA independent TAG synthesis

Removal of 18:1-OH from PC and formation of 18:1-OH-CoA may be critical steps in efficient tri-ricinolein synthesis in castor seeds. Lysophosphatidylcholine acyltransferase (LPCAT) catalyses these reactions but expression of candidate genes for this enzyme, 30170.m014002 or 30174.m008937, is broadly equivalent in all tissues (Table S3 - Acyl editing/alternative TAG synthesis). A better candidate for this reaction from these results is the membrane-bound O-acyltransferase (MBOAT) protein encoded by 30190.m011126, which has significantly raised FPKM values in endosperm. This gene belongs to a sub-family of MBOATs containing lysophospholipid acyltransferases with a preference for lysophosphatidylcholine [37], [38].

Formation of 18:1-OH-CoA may be catalysed by a lipase followed by ACS. Our data indicate that the LACS9 orthologue 29908.m006186 is probably important in acyl-CoA synthesis in castor seeds, but it is harder to identify a lipase that could produce 18:1-OH. Two triacylglycerol lipases, 29935.m000048 and 30183.m001305, have specific strong expression in endosperm with transcript levels increasing at the later developmental stage (Table S3 - Phospholipases/TAG lipases). Gene 29935.m000048 has previously been characterised as an acidic lipase of castor beans which is active on TAG [39] and although these two genes may not produce enzymes which remove *sn*-2 acyl groups from PC, their expression patterns suggests a role in seed development or lipid accumulation. An additional lipase candidate, 28885.m000108, was also strongly expressed in early endosperm. The protein encoded by this gene has homology to an Arabidopsis GDSL lipase (GLIP), but it remains to be determined if it has a phospholipase activity that may release modified fatty acids for TAG synthesis.

Analysis of the seven castor genes related to phospholipid:diacylglycerol acyltransferase (PDAT) shows *PDAT1A* and *PDAT2* (29912.m005286 and 29991.m000626 respectively) have the highest FPKM values and are expressed most strongly and specifically in the endosperm (Table S3 - Acyl editing/alternative TAG synthesis). It has recently been demonstrated that castor PDAT1A increases hydroxylated fatty acid amounts in transgenic seed oil [17], [18]. Two other enzymes in pathways of acyl-CoA independent TAG synthesis, CDP-choline:diacylglycerol choline-phosphotransferase (CPT) and phosphatidylcholine:diacylglycerol choline-phosphotransferase (PDCT) do not have such clear seed-specific expression as PDAT1A and 2. FPKM values for *PDCT* (29841.m002865) in the endosperm are only approximately double those in leaf or male flowers and *CPT* (30138.m003845) has equivalent, relatively low-level, expression in four of the samples (Table S3). This does not immediately suggest a major role for these two enzymes in tri-ricinolein synthesis and their importance for seed-oil synthesis in castor, as has been shown for PDCT in Arabidopsis [20], remains to be determined.

Selected genes encoding lipid metabolic and seed storage proteins were clustered based on tissue expression patterns (Figure S2). The major clade which includes oleate-12 hydroxylase contains 58 proteins, listed in Table S4. Most of the proteins discussed above have similar expression patterns to oleate-12 hydroxylase and are contained within it, the exceptions being GPAT9 (30122.m000357), LPCAT (30170.m014002 and 30174.m008937) and CPT (30138.m003845). Genes encoding these three proteins are more ubiquitously expressed and likely to be controlled by different regulatory systems.

#### 4. Expression of oleosin and storage-protein genes increases during seed development

Storage-protein genes exhibit strong induction of expression in the endosperm and their FPKM values are among the highest in this study (Table S3 - Seed storage proteins/ricin). Oil bodies in plant seeds are surrounded by oleosin proteins and transcription of two oleosins is high in the endosperm. In contrast to most of the lipid metabolic genes, expression of storage proteins and oleosins increases in the later stage of endosperm development. Our results show that transcriptional activation of lipid synthesis occurs very early in castor seed, before development of cellular endosperm, and does not coincide with high levels of ricin or storage protein expression.

#### 5. Comparison of RNA-Seq data and proteomic analysis of isolated endoplasmic reticulum from castor endosperm

Purification of the ER from castor endosperm (stage IV/V) enabled proteomic identification of membrane-bound TAG-synthetic enzymes in both acyl-CoA dependent and independent pathways [40]. Proteins encoded by several of the genes described above were detected in the ER, showing that gene products from loci with high endosperm FPKM values were present in this tissue. Based on the number of spectra detected in the MudPIT-type proteomic analysis, oleate-12 hydroxylase was substantially more abundant than oleate-12 desaturase in endosperm ER, reflecting the FPKM ratios observed for their genes. The putative cytochrome P450 (29791.m000529), a fatty acid ω-1 hydroxylase homologue, was also present in the ER. The most abundant ACS protein in enriched endosperm ER was the LACS9 homologue encoded by 29908.m006186. Other genes which are highly-expressed in the endosperm and encode proteins detected in enriched ER from this tissue are: *GPAT9* - 30122.m000357; *LPAT2* - 27810.m000646; *DGAT2* - 29682.m000581; *MBOAT* - 30190.m011126; *PDAT1A* - 29912.m005286; *PDCT* - 29841.m002865; GLIP-1 lipase - 28885.m000108 and triacylglycerol lipase - 30183.m001305. These results support the hypothesis that analysis of gene expression in endosperm and other castor tissues may help to pinpoint enzymes involved in an 18:1-OH specific TAG pathway.

### Transcripts for lipid biosynthesis are reduced in germinating seeds and β-oxidation transcripts and components of the glyoxylate cycle are prevalent

Germinating seeds degrade lipids derived from storage TAG and convert them into sugars which support early development [41]. TAGs are first hydrolysed by lipases, releasing free fatty acids which enter peroxisomes. Here they undergo β-oxidation to form acetyl-CoA which is converted to oxaloacetate by the glyoxylate pathway. Following export from the peroxisome oxaloacetate is used in gluconeogenic reactions to synthesise carbohydrates. It is not therefore unexpected that expression of fatty acid synthetic (FAS and ACCase) genes is greatly decreased in germinating compared to developing castor seeds (Table S3 - Fatty acid synthesis). Amongst other lipid metabolic genes in castor, β-oxidation pathway components including 3-hydroxyacyl-CoA dehydrogenase, 3-ketoacyl-CoA thiolase B, acyl-CoA oxidase and acyl-CoA dehydrogenase are highly expressed in germinating seed, together with proteins involved in fatty acid desaturation in the ER: FAD2 and 3, cytochrome b5 and NADH-cytochrome b5 reductase (Table S5).

It is evident that differential regulation of ACS genes occurs in developing and germination seeds (Table S3 - Acyl-CoA activation). Transcription of *ACS2*, 29844.m003365, is strongly induced in germinating seed and it is clearly the dominant isoform expressed in this tissue, followed by 29732.m000322. In contrast, the FPKM value for the most highly expressed LACS in developing seed, 29908.m006186, is dramatically reduced during germination. This pattern is analogous to that seen in Arabidopsis, where two specific LACS isoforms are associated with peroxisomal fatty acid activation during germination (LACS6 and 7) and different enzymes (LACS1 and LACS9) are induced during oil synthesis in developing seeds.

Significant differences in expression of lipases between these tissues are also apparent. Transcripts for the three lipases induced in seed development (29935.m000048, 30183.m001305 and 28885.m000108) are essentially absent in the germinating seed and expression of others, such as 29884.m000182, 29884.m000183, 30167.m000881 and 28470.m000422 is increased (Table S3 - Phospholipases/TAG lipases). The latter is a homologue of the SDP1 lipase required for TAG breakdown and normal germination in Arabidopsis seeds [42] but the substrates of these castor lipases are not proven.

One of the most abundant transcripts in germinating seeds is that coding for isocitrate lyase (Table S6) which generates glyoxylate and succinate from isocitrate as part of the glyoxylate cycle. Other members of the glyoxylate cycle such as malate synthase, malate dehydrogenase and aconitase are also highly expressed in this tissue. Full lists of genes which have sequencing reads mapped to them in each tissue are in Table S7.

### Comparison of transcripts in developing seed and leaf

Transcripts encoding photosynthetic proteins for both light-harvesting and Calvin cycle reactions are abundant in leaf RNA (Table S6). No lipid metabolic pathway is obviously up-regulated in leaf (such as occurs with β-oxidation in germinating seed) and FPKM values for almost all of the fatty acid biosynthetic enzymes that are expressed in developing seed are substantially lower in leaf. A leaf-specific acyl carrier protein (30147.m014425) can easily be identified (Table S3 – Fatty acid synthesis) and increased expression of both palmitoyl-ACP thioesterase (29848.m004677) and plastidial desaturases (29666.m001456, 29696.m000105 and 28176.m000273) reflects an increased demand for photosynthetic membrane fatty acids. Unsurprisingly, read counts for oleate-12 hydroxylase are orders of magnitude lower in leaf than seed.

Three GPAT genes have higher expression in the leaf than the seed, one of which is the plastidial GPAT. The other two are homologues of Arabidopsis *GPAT4* and *6* which were recently shown to be required for synthesis of cutin, a polyester found in the extracellular surface barrier of leaves and other plant aerial parts [43]. Transcript levels of three TAG-synthetic acyltransferases are noticeably reduced in leaf compared to seed. Expression of two PDATs, 29912.m005286 and 29991.m000626, is low in leaf and the FPKM value for DGAT2 is reduced by 7-fold in this tissue. These three enzymes have expression patterns which suggest they are important in seed oil metabolism. Finally gene 29889.m003411, a soluble DGAT/wax synthase, is up-regulated in leaf although it is currently unclear whether its product is involved with TAG or wax synthesis.

### Differential expression of lipid metabolic gene isoforms in developing endosperm and male developing flowers which are producing pollen

Lipids play a number of roles in the formation and viability of pollen. TAG is present in pollen and it is thought mobilization and β-oxidation of its constituent fatty acids provides an energy source for pollen tube growth during fertilisation [44]. Lipids such as sterol esters and TAG accumulate in the tapetal cell layer which surrounds developing pollen grains and components such as sterol esters are released and incorporated into the pollen surface when the tapetum breaks down [45], [46]. Precursors required for sporopollenin and tryphine formation, derived mainly from saturated precursors such as long-chain fatty acids or long aliphatic chains, are also provided by the tapetum [47]. The importance of lipid synthesis to pollen development is shown by the fact that knockout of either trienoic fatty acid or TAG synthesis results in non-viable pollen [48], [49]

Castor pollen and male flowers contain TAG which is enriched in 16:0, 18:2 and 18:3 fatty acids. Developing male flowers were selected as a negative type of control to aid identification of genes important for tri-ricinolein synthesis in the endosperm. Clearly there is major difference in expression of the oleate-12 hydroxylase gene, with no reads mapped in male flowers (Table S3), but differential regulation of other lipid metabolic genes is also apparent.

Expression of FAS and ACCase components is generally reduced in male flowers compared to the endosperm, although exceptions are the two FATB thioesterases which terminate fatty acid synthesis at 16 carbons (Table S3 - Fatty acid synthesis). One of the stearoyl-ACP desaturase genes (29929.m004514) is the most highly expressed fatty acid synthetic gene in male flowers, although it is not specific for this tissue and is strongly induced in endosperm as well. Elevated transcription of other desaturases is apparent. Expression of both ω-3 (Fad3) and ω-6 (Fad2) ER desaturases and a homologue of Arabidopsis *FAD5*, 29841.m002863, is also up-regulated in this tissue (Table S3 - Fatty acid modification), perhaps to be expected given the high proportion of polyunsaturated (18:2 and 18:3) fatty acids present (Table 2).

Two ACS isoforms, the *LACS1* homologue 30076.m004616 and ACS1, 30190.m10831, have higher FPKM values in male flowers, with expression of the former specifically induced in this tissue (Table S3 – Acyl-CoA activation). ACS enzymes encoded by these genes may important for synthesising pollen lipids or surface components and a requirement for specific ACS isoforms in pollen coat formation has been demonstrated in Arabidopsis [50]. It is notable that transcription of the LACS9 homologue 29908.m006186, which is most highly expressed in endosperm, is greatly reduced in male flowers.

A homologue of Arabidopsis *GPAT6* (29969.m000267) has the highest expression of any TAG-synthetic gene with an FPKM value of 566 in male flowers. GPAT6 is involved in cutin biosynthesis and tapetum development in Arabidopsis [51], [52]. Another acyltransferase with strongly elevated transcription only in male flowers, 29637.m000766 (designated as phosphatidylcholine acyltransferase in the castor database) is homologous to phospholipid sterol O-acyltransferases and is likely to be required for sterol ester synthesis in the tapetum. Several genes discussed in previous sections, with high FPKM values in the endosperm and possible roles in TAG synthesis, have reduced expression in male flowers (Table S3). This is particularly true for PDAT1 and 2, but DGAT2, MBOAT protein (30190.m01126) and PDCT have 4.6, 5.4 and 2.4-fold respectively higher transcript levels in the endosperm which suggests these enzymes may be important for tri-ricinolein synthesis in endosperm.

Tissue specific expression of four TAG lipases is evident in male flowers and endosperm (Table S3 Phospholipases/TAG lipases). Two of these, 29646.m001089 and 29646.m001091, are strongly activated in male flowers and may be associated with mobilization of pollen oil reserves during tube growth and fertilisation. Degradation of TAG during anther development in Brassicaceae has been reported [46] and lipases specifically expressed in male flowers could play a role in this process, releasing lipids required for pollen maturation. Transcription of TAG lipases 29935.m000048 and 30183.m001305, which were strongly transcribed in the endosperm, was not detected in male flowers. Although this is consistent with a role in tri-ricinolein synthesis, and they may be involved in re-modelling of TAGs after synthesis in developing castor bean [53], the function of these lipases remains unclear.

Transcription of genes encoding enzymes which elongate fatty acids and make wax, cutin and alkane precursors is increased in male flowers. 3-ketoacyl-CoA synthase (KCS) enzymes catalyse the initial condensation steps in microsomal fatty acid elongation. *KCS* transcripts are among the most abundant from lipid metabolic genes in male flowers (Table S5) and homologues of Arabidopsis KCS 10 and 21 (29726.m004062 and 29777.m000271) are only expressed in this tissue (Table S3 – FA elongation/wax synthesis). Other components are required to complete an elongation cycle and increased expression of microsomal 3-ketoacyl-CoA reductase and 3-hydroxyacyl-CoA dehydratase isoforms also occurs. Strong induction of 29709.m001216, a homologue of Arabidopsis *CER1* required for wax synthesis and necessary for pollen development [54], and expression of two proposed alcohol O-fatty-acyltransferases (wax synthases), 27613.m000611 and 29629.m001370, reflect a demand for surface and cuticular lipids in developing male flowers. Homologues of several Arabidopsis lipid metabolic genes, with proven roles in pollen formation and male fertility, are significantly up-regulated in male flowers compared to the endosperm (Table S8).

### Expression of castor transcription factor genes

Transcription factors (TFs) are DNA binding proteins that regulate gene expression which are encoded by distinct and conserved multigene families in plants. The number of genes and gene transcripts encoding members of a particular TF family may differ between plant species and specific tissues on account of specific function(s) and/or evolutionary expansion/contraction. The expression of TFs in plant seeds is of substantial interest as a route to identify potential master regulators that control genes with roles in pathways of reserve accumulation, especially lipids. Analysis of microarray datasets indicates that a number of plant lipid metabolism genes are transcriptionally co-regulated [55], [56], but specific factors controlling gene transcription for TAG metabolism in castor and other oilseeds remain to be uncovered.

A complex network, including several TFs, determines aspects of seed and embryo development [57]. In *Arabidopsis thaliana*, LEAFY COTYLEDON1 (LEC1) and LEC1-LIKE (L1L) are key regulators of fatty acid biosynthesis. The transcription factor WRINKLED1 (WRI1), which belongs to the APETALA2-ethylene responsive element-binding protein (AP2-EREBP) family, controls fatty acid, but not TAG, synthesis via LEC2 and possibly LEC1 [58] and it is likely that additional TFs are involved. Complete regulatory mechanisms controlling other lipid metabolic pathways, such cuticular wax and cutin biosynthesis, are also not known. The Arabidopsis AP2-EREBP TF WIN1/SHN1 activates cuticular wax biosynthesis [59] and genes involved in cutin biosynthesis such as *GPAT4* and possibly *LACS2* [60] but much remains to be discovered.

We analysed expression of TF in the five castor tissues. Castor protein sequences were searched against the Pfam library of HMMs to identify Pfam domains. A custom perl script was used to identify and classify transcription factors according to rules utilised in the plant transcription factor database PlnTFDB (http://plntfdb.bio.uni-potsdam.de/v3.0/) [61]. A total of 1310 TFs were identified in the castor genome, which can be divided into 60 families (Table S9, TF families sheet). The largest four TF families were AP2-EREBP, bHLH, MYB-related and NAC domain, with 115 (8.8%), 103 (7.9%), 97 (7.4%) and 95 (7.2%) genes respectively. BLAST P alignment against the TAIR9.0 database (expect score 1×10^−5^) detected Arabidopsis homologues for 1289 out of these 1310 castor TF. Only 919 Arabidopsis TF were represented in these alignments versus 1289 castor proteins, which may reflect their evolutionary relationship.

Transcripts were detected in at least one of the RNA samples for 1060 castor TF, 157 of which were specific for one tissue (Table S9, TF expression sheet). Expression of 41 TF was confined to the endosperm. In order to associate TF expression to 18:1-OH and tri-ricinolein metabolism, data for TFs in endosperm stages and male developing flowers were compared (Table S9, Endosperm – flower TF sheet). In addition to the 41 TF which were only transcribed in endosperm, 26 with endosperm FPKM values >5 were not expressed in flowers and a further 31 had this FPKM cut-off and (FPKM EII-III/FPKM Male flowers) values >10. Investigation of the targets for some of these TF may be important to determine factors linked to high levels of ricinoleic acid in castor oil.

### Concluding statements

In this study we have examined the fatty acid composition of a variety of castor tissues and shown that ricinoleic acid (18:1-OH) is present in both developing endosperm and cotyledons but absent from male developing flowers or pollen. Triacylglycerol (TAG) accumulates in both of these tissues, but its fatty acid composition is different to TAG synthesised in developing castor seeds. TAG in the seed-endosperm predominantly contains 18:1-OH, but this is absent from pollen and flower TAG which is instead mainly composed of 16:0, 18:2 and 18:3 fatty acids. Analysis of the acyl-CoAs in developing endosperm revealed that 18:1-OH-CoA is not the major acyl-CoA species. This is consistent with high levels of 18:1-OH accumulation in TAG being driven by one, or a combination of, the following: [a] restricted access to different *in vivo* pools of acyl-CoAs, [b] strict metabolic channelling and [c] substrate selectivity of enzymes involved in their biosynthesis.

TAG biosynthesis in castor endosperm occurs in the endoplasmic reticulum (ER). A previous proteomic study from our group identified proteins in purified endosperm ER and detected TAG-biosynthetic proteins in developing oil-seeds for the first time. We also reported quantitative RT-PCR data comparing expression in seed and leaf of selected genes potentially involved in tri-ricinolein biosynthesis. In order to extend that study and gain a better understanding of gene expression in castor, we performed RNA-Seq studies in a variety of tissues. This report focuses on genes associated with lipid metabolism and a key aim was comparison of transcription in endosperm and male developing flowers. We believe it unlikely that the different TAG compositions in these tissues is solely due to oleate-12 hydroxylase activity, since expression of this enzyme in a number of plant species does not result in synthesis of high 18:1-OH TAG. Comparison of expression between tissues allowed identification of candidate genes which may be important for tri-ricinolein synthesis in castor seed in addition to the oleate-12 hydroxylase – Table 3. Gene products from nine of these eleven genes were detected in purified endosperm ER, the site of TAG synthesis. Expression of two of these genes, *DGAT2* and *PDAT1A*, in plants expressing oleate-12 hydroxylase elevated 18:1-OH incorporation into seed oils and co-expression of additional genes in Table 3 might increase this level further. We are currently performing pull-down and native blue PAGE experiments to identify interacting partners and complexes involved in TAG synthesis in endosperm ER, which should advance understanding of metabolic compartmentalisation in this tissue. A combination of these two approaches could lead to higher 18:1-OH levels in future transgenic plants.

**Table 3:** Candidate genes that may be important in tri-ricinolein synthesis based on RNA-Seq data.

Gene ID	Gene Identifier	Detected in ER
27810.m000646	Lysophosphatidic acid acyltransferase (LPAT2)	x
28885.m000108	GLIP1 carboxylesterase/lipase	x
29682.m000581	Type 2 diacylglycerol acyltransferase (DGAT2)	x
29791.m000529	Cytochrome P450, Putative	x
29841.m002865	Phosphatidylcholine diacylglycerol cholinephosphotransferase (PDCT)	x
29908.m006186	Long-Chain-Fatty-Acid CoA Ligase, Putative (ACS9)	x
29912.m005286	Phospholipid:diacylglycerol acyltransferase 1 (PDAT1A)	x
29935.m000048	Triacylglycerol Lipase, Putative	
29991.m000626	Phosphatidylcholine:Diacylglycerol Acyltransferase 2 (PDAT2)	
30183.m001305	Triacylglycerol Lipase, Putative	x
30190.m011126	O-Acyltransferase (Membrane Bound) Domain Containing Protein	x

A milestone in castor research was the recent sequencing of its genome [22]. Here we report transcriptome analysis of five tissues from this species, using a paired end sequencing approach with read lengths of 120 bases. Previous castor expression studies [29], [30] (http://www.ncbi.nlm.nih.gov/sra accession SRX007402 to SRX007408) were limited to developing seeds. High-quality RNA-Seq data allows identification and accurate quantification of transcription and the results presented here supplement information on expression of lipid metabolic genes in soybean [62] *Jatropha curcus* [63], bitter melon [64] and Arabidopsis [65].

Our data provides sequence information that could be used to update the current castor genome assembly and identify new genes that were not originally annotated by largely automated processes. To indicate the potential for this approach, RNA-Seq reads were aligned to the reference genome and output from each of the five tissue samples separately assembled using the Cufflinks package. Resulting assemblies were then merged and compared to the JCVI reference annotation. In total the RNA-Seq reads assembled into 75090 transcripts corresponding to 29759 ‘genes’ which compares to the 31221 genes in version 0.1 of the JCVI assembly. Of the 29759 cufflinks genes, 2847 were located intergenic to the JCVI annotation and hence may represent novel genes. A total of 218147 splice junctions were identified from mapping the RNA-Seq reads, with 112337 supported by at least 10 reads. Comparison of these junctions to the JCVI annotation shows over 30000 are distinct, with ∼4000 mapping intergenic. While the Cufflinks assemblies will be incomplete and contain errors, such as incorrect merges and retained introns, it is clear that integrating the RNA-Seq data into the JCVI gene build would allow the annotation of several thousand alternative splice variants and potentially several hundred novel genes. Such integration is outside the scope of this work, but the RNA-Seq data reported here clearly provides a valuable resource for evaluation of current gene models (http://castorbean.jcvi.org) and investigation of alternative splicing or single nucleotide polymorphisms in castor.

## Materials and Methods

### Plant growth and tissue collection

Castor (*Ricinus communis*) variety 99N89I was grown in John Innes No.3 compost with a 16/8 h light/dark cycle at 23°C and 18°C. Plants were watered daily and inflorescences tagged on emergence of the first female flowers. Complete flowering stems were removed 15 to 20 or 25 to30 days after flowering and endosperm samples removed for immediate freezing in liquid nitrogen. Fruit were cut transversely and the endosperm appearance enabled developmental staging according to Greenwood and Bewley [25]. Cotyledons for lipid analysis were dissected out of whole developing beans at stage V/VI. All material collected for RNA preparation was frozen in liquid nitrogen and stored at −80°C. For RNA extraction, equal amounts of endosperm at stages II/III (endosperm free-nuclear stage) or stages V/VI (onset of cellular endosperm development) were pooled from at least five different castor plants. Expanding true leaves, appearing after the first cotyledons and leaf-pair, were harvested when they were 10–15 cm in size. Germinating seed (cotyledon) tissue was obtained by soaking dry beans in running water overnight followed by germination in the dark for 3 days in moist vermiculite at 30°C and material from >30 separate seeds was pooled. Developing male developing flowers were harvested from at least 10 castor plants. The perianth of the male flower (comprised of sepals) was removed using a razorblade and reproductive organs, including pollen and anthers, collected.

### Acyl-CoA analysis

Endosperm samples were pooled according to developmental stage from a number of different inflorescences. Mixed samples were ground in a pestle and mortar and 10–20 mg transferred into chilled eppendorfs. Acyl-CoA extraction and measurement were as reported previously using the modified mobile phase composition for extended HPLC separation [24], [66].

A ricinoleoyl-CoA standard to confirm peak identity was synthesised from an ammonium salt of ricinoleic acid (18:1-OH). This was made by adding 0.5 ml 2 M NH_3_ solution to 10 mg 18:1-OH (Sigma R7257), incubating at 60°C for 30 minutes and adding 0.5% Triton X-100 after cooling and removal of NH_3_ under nitrogen. The acyl-CoA was made in a 1 ml reaction containing 0.1 M Tris/HCl pH 7.5, 10 mM MgCl_2_, 10 mM ATP, 1 mg 18:1-OH, 0.25% Triton X-100, 10 mM CoASH and 1 U acyl-CoA synthetase (Sigma). After incubation for 3 hour at 30°C, 18:1-OH-CoA was purified using a 1 ml Sep-Pak C18 Plus column (Waters) which had been equilibrated with 6 ml methanol and 6 ml Tris/HCl pH 7.5. The reaction was loaded and the column washed with 1.5 ml Tris/HCl pH 7.5 and 1.5 ml 50% MeOH before elution with 5 ml HPLC-grade MeOH. Progression of synthesis and eluted products were checked by TLC on silica K6F plates (Whatman) developed with butanol/acetic acid/water (5∶2∶3). Methanol was removed by vacuum centrifugation, the 18:1-OH-CoA resuspended in 10 mM NaOAc pH5.9 and concentration determined by A_260_. Samples ranging from 1 to 100 pmoles were analysed as described above.

### Lipid analysis

Lipid extraction from castor tissues used the Folch method [67]. Pollen was incubated in chloroform/methanol 2∶1 at 50°C for 10 minutes before extraction at room temperature, while other tissues were homogenised in this solvent to start the procedure. Aliquots of the final extracts were dried and fatty acid composition determined by fatty acid methyl ester (FAME) analysis as described [5].

Total lipids were prepared from 4.5 ml volume pollen using the Folch method. Inhibition of lipases with isopropanol before extraction did not alter the lipid profiles observed. Lipid-X was purified with two Flash chromatography runs on silicic acid columns (100–200 mesh Sigma) developed with hexane/diethylether/acetic acid (70∶30∶1): see Figure S1. Eluting fractions were analysed by thin layer chromatography on silica gel 60 aluminium-backed plates (Merck) developed in the solvent system above and fractions containing lipid-X pooled. FAME analysis was as for the tissue lipid extracts. For analysis by mass spectrometry, purified lipids were re-suspended in methanol/chloroform (4∶1) and 2.5 mM lithium acetate added before static infusion into a hybrid quadropole-TOF mass spectrometer (QStar Pulsar *i*, Applied Biosystems). Selected ions were fragmented by collision induced dissociation following introduction of nitrogen and products analysed by time of flight.

### Whole transcriptome cDNA library construction and sequencing

The castor tissues for RNA-Seq analyses were ground to powder in liquid nitrogen and total RNA was prepared using a minor modification of the instructions of the ToTALLY RNA Kit (Ambion). To selectively deplete abundant rRNA molecules, mRNA was enriched using the MicroPoly(A)Purist Kit with two rounds of oligo dT purification (Ambion). The mRNA quality was checked on a Nano chip of the Bioanalyser 2100 (Agilent) and the quantity measured with the Qubit RNA kit and Qubit fluorometer (Invitrogen). Transcriptome libraries were constructed using the Illumina mRNA Seq sample preparation kit with some modifications. In brief, purified mRNA was fragmented by addition of 5× fragmentation buffer (Illumina, Hayward, CA) and was heated for 2 min at 94°C in a thermocycler. A shorter fragmentation time was used to yield library size of 350–400 bp. First strand cDNA was synthesised using random primers to eliminate the general bias towards 3′ end of the transcript. Second strand cDNA synthesis was done by adding GEX second strand buffer (Illumina, Hayward, CA), dNTPs, RNaseH and DNA polymerase I followed by incubation for 2.5 h at 16°C. Second strand cDNA was further subjected to end repair, A-tailing, and adapter ligation in accordance with the manufacturer supplied protocols. Purified cDNA templates were enriched by 15 cycles of PCR for 10 s at 98°C, 30 s at 65°C, and 30 s at 72°C using PE1.0 and PE2.0 primers and with Phusion DNA polymerase (Illumina, Hayward, CA). The samples were cleaned using QIAquick PCR purification columns and eluted in 30 µl EB buffer as per manufacturer's instructions (QIAGEN, CA). Purified cDNA libraries were quantified using Bioanalyser DNA 100 Chip (Agilent). All libraries were sequenced on the Illumina Genome Analyzer II as paired end 120 bp reads following the manufacturer's recommendations.

### Data processing and analysis of Illumina RNA-Seq reads

The Illumina processing pipeline version 1.3 was employed for processing of raw images to make base calls and generate sequence reads, the default quality filter (CHASTITY≥0.6) was applied. FASTQ files were generated for each of the five tissues. The number of paired end reads that passed the quality filter for each tissue is shown in Table S1 total reads. Sequence data has been submitted to the ENA sequence read archive (SRA) under accession ERA047687.

### Mapping of RNA-Seq reads to castor genome

Reads for each sample were aligned to the TIGR castor bean WGS assembly release 0.1 (http://castorbean.jcvi.org/index.php) using Tophat v1.2.0 [68]. Tophat aligns RNA-Seq data to the genome first using Bowtie [69] to identify coverage islands. In this initial step reads are split into smaller segments and mapped independently. The resulting coverage islands are used to generate a junction database that is supplemented with junctions directly inferred from paired end data. Additionally we supplied Tophat with all annotated junctions in the TIGR castor bean genome release 0.1 though the –G parameter. Tophat was run with default alignment parameters, allowing multiple mappings per read (40 default) and 2 mismatches in each segment alignment, the max intron length was changed to 20,000 bp.

### Differential expression testing using Cuffdiff

Differential expression testing was carried out using the Cuffdiff program from the Cufflinks package version 0.9.3 [28]. Transcript abundance was determined for the gene models annotated in the TIGR castor bean genome release 0.1 using the Tophat RNA-Seq BAM alignments for each of the five tissue samples. Cuffdiff reports an expression value for each transcript and gene in Fragments Per Kilobase of exon model per Million mapped fragments (FPKM). A test statistic is also calculated which after Benjamini-Hochberg correction for multiple-testing [70] was used to determine significant changes in expression between each pair of samples.

## Supporting Information

Figure S1 **Purification of lipid for GC and MS analysis.**
(TIF)

Figure S2 **Heatmap of selected lipid metabolism and storage protein genes differentially expressed in five castor tissue samples.**
(PDF)

Table S1 **Summary of sequence read mapping.**
(DOC)

Table S2 **Expression values of castor gene models and significant differences between castor tissues.**
(XLS)

Table S3 **Read counts of candidate genes for fatty acid, glycerolipid and wax metabolism and for storage proteins.**
(XLS)

Table S4 **Genes in the heatmap made from lipid synthetic and storage proteins that are co-expressed with oleate hydroxylase in Ricinus tissues.**
(XLS)

Table S5 **Expression of lipid metabolic genes in castor germinating seeds, developing endosperm and male flowers.**
(XLS)

Table S6 **Castor genes with the highest FPKM values in the five castor tissues.**
(XLS)

Table S7 **Castor gene models ordered on FPKM values in individual tissues.**
(XLS)

Table S8 **Lipid metabolism genes with proven roles in male gametophyte and floral development.**
(DOC)

Table S9 **Expression of castor transcription factors.**
(XLS)
