# Supplementary figures and images for: Tissue-Specific Whole Transcriptome Sequencing in Castor, Directed at Understanding Triacylglycerol Lipid Biosynthetic Pathways
Source: PLoS One. 2012 Feb 3;7(2):e30100. doi: 10.1371/journal.pone.0030100 (PMC3272049; doi:10.1371/journal.pone.0030100)

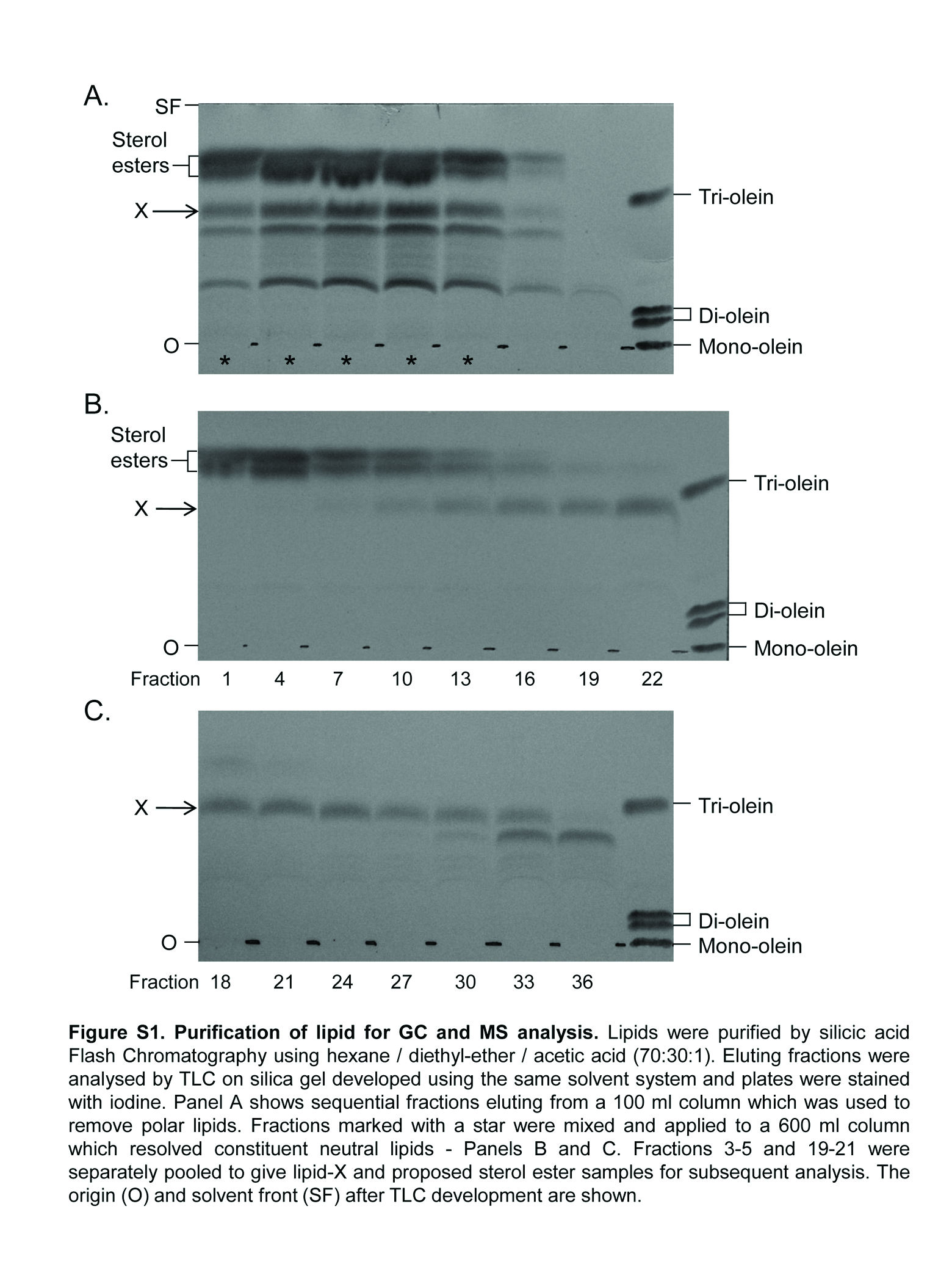

Supplement: Figure S1 — Purification of lipid for GC and MS analysis. (TIF) [file pone.0030100.s001.tif]
