# Supplementary material for: Tissue-Specific Whole Transcriptome Sequencing in Castor, Directed at Understanding Triacylglycerol Lipid Biosynthetic Pathways
Source: PLoS One. 2012 Feb 3;7(2):e30100. doi: 10.1371/journal.pone.0030100 (PMC3272049; doi:10.1371/journal.pone.0030100)
Supplement: Figure S2 — Heatmap of selected lipid metabolism and storage protein genes differentially expressed in five castor tissue samples. (PDF) [file pone.0030100.s002.pdf]

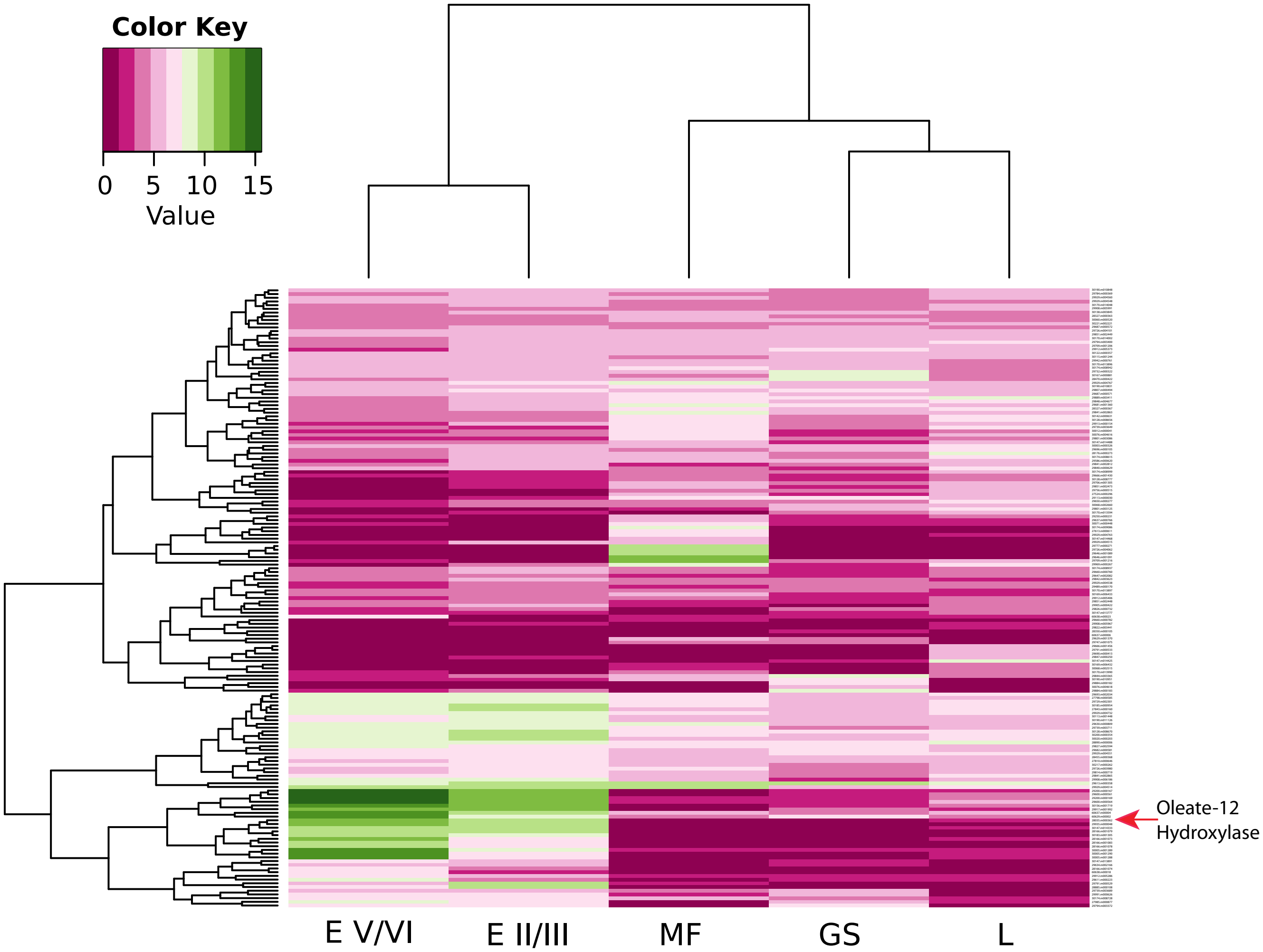

Figure S5. Heatmap of selected lipid metabolism and storage protein genes differentially expressed in five castor tissue samples. The heatmap was generated in R from expression data of the castor gene models in Supplementary Table S3 using the heatmap.2 function in the gplots CRAN library and FPKM normalized log2 transformed values with hierarchical clustering represented by the colour scale (1–>15). Green indicates high expression, light-green/pink indicates intermediate expression and purple indicates low expression. Tissue samples were: developing endosperm stage II/III, E II/III; developing endosperm stage V/VI, E V/VI; developing male flowers, MF; germinating seed, GS; leaf, L.
