# Supplementary material for: Tissue-Specific Whole Transcriptome Sequencing in Castor, Directed at Understanding Triacylglycerol Lipid Biosynthetic Pathways
Source: PLoS One. 2012 Feb 3;7(2):e30100. doi: 10.1371/journal.pone.0030100 (PMC3272049; doi:10.1371/journal.pone.0030100)
Supplement: Table S1 — Summary of sequence read mapping. (DOC) [file pone.0030100.s003.doc]

**Table S1. Summary of sequence read mapping**

|  | Endosperm II/III | Endosperm V/VI | Germinating seed | Leaf | Male flowers |
| --- | --- | --- | --- | --- | --- |
| Total reads | 21,858,754 | 14,326,641 | 17,374,537 | 19,322,724 | 17,869,625 |
| Total number of positions mapped | 36,412,207 | 25,447,316 | 29,038,424 | 32,267,706 | 28,774,682 |
| Total Reads mapped | 19,924,436 | 13,130,084 | 15,892,926 | 15,749,361 | 16,193,189 |
| Percentage of reads mapped | 91 | 92 | 91 | 82 | 91 |
