# Supplementary material for: Tissue-Specific Whole Transcriptome Sequencing in Castor, Directed at Understanding Triacylglycerol Lipid Biosynthetic Pathways
Source: PLoS One. 2012 Feb 3;7(2):e30100. doi: 10.1371/journal.pone.0030100 (PMC3272049; doi:10.1371/journal.pone.0030100)
Supplement: Table S8 — Lipid metabolism genes with proven roles in male gametophyte and floral development. (DOC) [file pone.0030100.s010.doc]

Table S8. Lipid metabolism genes with proven roles in male gametophyte and floral development

| *A. thaliana* Gene ID | *R. communis* Gene ID | Gene description^a^ | Function | Ref. | E II/III | MF |
| --- | --- | --- | --- | --- | --- | --- |
| At1g68530 | 27524.m000296 | *CUT1* *(KCS6)* acyltransferase, putative | Cuticular wax biosynthesis, pollen fertility | [1] | 0.0 | 37.1 |
| At1g02205 | 29709.m001216 | *CER1* Sterol Desaturase, Putative | Epicuticular wax biosynthesis, pollen fertility | [2] | 1.4 | 4313.2 |
| At5g57800 | 29783.m000308 | *WAX2 or FLP1* Sterol Desaturase, Putative | Pollen sperm cell differentiation, fertility, sporopollenin synthesis | [3,4] | 7.4 | 485.0 |
| At1g69500 | 29813.m001518 | *CYP704B1* cytochrome P450, putative | Sporopollenin synthesis | [5] | 0.8 | 71.9 |
| At3g11980 | 28516.m000032 | *MS2 (fatty acyl reductase)* Male sterility protein, putative | Male gametogenesis | [6] | 2.1 | 10.1.4 |
| At5g13390 | 30128.m008576 | *NEF1* conserved hypothetical protein | Tapetum plastid lipids, exine formation, male sterility | [7] | 44.4 | 22.0 |
| At1g01280 | 29742.m001406 | *CYP703A2 (DEX2)* flavonoid 3-hydroxylase,putative | Sporopollenin synthesis | [8] | 0.5 | 207.5 |
| At2g19690 At4g29460 At4g29470 | 29912.m005406 | *PLA2-β, PLA2-γ, PLA2-d* Phospholipase A23 | Pollen development, germination, tube growth | [9] | 9.0 | 18.8 |
| At2g29980 At3g11170 At5g05580 | 29681.m001360 28176.m000273 29814.m000719 | *FAD3, FAD7, FAD8* Omega-3 fatty acid desaturase | JA synthesis, anther and pollen development | [10] | 45.3 29.8 92.5 | 224.1 25.2 59.5 |
| At2g38110 | 29969.m000267 | *GPAT6* ER glycerol-3-phosphate acyltransferase (GPAT) | Floral cutin synthesis; tapetum and stamen development; fertility | [11,12] | 13.4 | 565.6 |
| At1g06520 | 28350.m000105 | *GPAT1* ER glycerol-phosphate acyltransferase (GPAT) | Tapetum differentiation, male fertility | [13] | 0.0 | 0.0 |
| At2g19450 | 29912.m005373 | *DGAT1* Type I diacylglycerol acyltransferase | Normal pollen and seed development | [14] | 26.9 | 53.5 |
| At5g13640 | 29706.m001305 | *PDAT1* Phospholipid:diacylglycerol acyltransferase1 | Normal pollen and seed development | [14] | 4.9 | 59.1 |
| At2g47240 | 30076.m004616 | *LACS1* Acyl-CoA synthetase | Tryphin biosynthesis, pollen coat formation, male sterility, flower development |  | 7.7 | 148.5 |
| At4g23850 | 30190.m010831 | *LACS4* Acyl-CoA synthetase (ACS1) | Tryphin biosynthesis, pollen coat formation, male sterility | [15] | 69.6 | 179.1 |
| At1g62940 | 30131.m006921 | *ACOS5* AMP dependent CoA ligase | Pollen development, sporopollenin synthesis | [17] | 21.8 | 250.0 |

FPKM values for developing endosperm stage II/III (E II/III) and pollen-producing developing male flowers (MF) are shown for castor gene models orthologous to Arabidopsis lipid metabolic genes with proven functional roles in pollen and flowers. ^a^ Gene descriptions in italics are from Arabidopsis and in non-italics from the castor database.

1. Millar AA, Clemens S, Zachgo S, Giblin EM, Taylor DC, et al. (1999) CUT1, an Arabidopsis gene required for cuticular wax biosynthesis and pollen fertility, encodes a very-long-chain fatty acid condensing enzyme. Plant Cell 11: 825-838.

2. Aarts MG, Keijzer CJ, Stiekema WJ, Pereira A (1995) Molecular characterization of the CER1 gene of Arabidopsis involved in epicuticular wax biosynthesis and pollen fertility. Plant Cell 7: 2115-2127.

3. Chen X, Goodwin SM, Boroff VL, Liu X, Jenks MA (2003) Cloning and characterization of the WAX2 gene of Arabidopsis involved in cuticle membrane and wax production. Plant Cell 15: 1170-1185.

4. Ariizumi T, Hatakeyama K, Hinata K, Sato S, Kato T, et al. (2003) A novel male-sterile mutant of Arabidopsis thaliana, faceless pollen-1, produces pollen with a smooth surface and an acetolysis-sensitive exine. Plant Mol Biol 53: 107-116.

5. Dobritsa AA, Shrestha J, Morant M, Pinot F, Matsuno M, et al. (2009) CYP704B1 Is a long-chain fatty acid omega-hydroxylase essential for sporopollenin synthesis in pollen of Arabidopsis. Plant Physiol 151: 574-589.

6. Aarts MG, Hodge R, Kalantidis K, Florack D, Wilson ZA, et al. (1997) The Arabidopsis MALE STERILITY 2 protein shares similarity with reductases in elongation/condensation complexes. Plant J 12: 615-623.

7. Ariizumi T, Hatakeyama K, Hinata K, Inatsugi R, Nishida I, et al. (2004) Disruption of the novel plant protein NEF1 affects lipid accumulation in the plastids of the tapetum and exine formation of pollen, resulting in male sterility in Arabidopsis thaliana. Plant J 39: 170-181.

8. Morant M, Jorgensen K, Schaller H, Pinot F, Moller BL, et al. (2007) CYP703 is an ancient cytochrome P450 in land plants catalyzing in-chain hydroxylation of lauric acid to provide building blocks for sporopollenin synthesis in pollen. Plant Cell 19: 1473-1487.

9. Kim HJ, Ok SH, Bahn SC, Jang J, Oh SA, et al. (2011) Endoplasmic reticulum- and Golgi-localized phospholipase A2 plays critical roles in Arabidopsis pollen development and germination. Plant Cell 23: 94-110.

10. McConn M, Browse J (1996) The critical requirement for linolenic acid is pollen development, not photosynthesis, in an Arabidopsis mutant. Plant Cell 8: 403-416.

11. Li-Beisson Y, Pollard M, Sauveplane V, Pinot F, Ohlrogge J, et al. (2009) Nanoridges that characterize the surface morphology of flowers require the synthesis of cutin polyester. Proc Natl Acad Sci USA 106: 22008-22013.

12. Li XC, Zhu J, Yang J, Zhang GR, Xing WF, et al. (2011) Glycerol-3-phosphate acyltransferase 6 (GPAT6) is important for tapetum development in Arabidopsis and plays multiple roles in plant fertility. Molecular Plant doi:10.1093/mp/ssr057.

13. Zheng ZF, Xia Q, Dauk M, Shen WY, Selvaraj G, et al. (2003) Arabidopsis AtGPAT1, a member of the membrane-bound glycerol-3-phosphate acyltransferase gene family, is essential for tapetum differentiation and male fertility. Plant Cell 15: 1872-1887.

14. Zhang M, Fan J, Taylor DC, Ohlrogge JB (2009) DGAT1 and PDAT1 acyltransferases have overlapping functions in Arabidopsis triacylglycerol biosynthesis and are essential for normal pollen and seed development. Plant Cell 21: 3885-3901.

15. Jessen D, Olbrich A, Knufer J, Kruger A, Hoppert M, et al. (2011) Combined activity of LACS1 and LACS4 is required for proper pollen coat formation in Arabidopsis. Plant J doi: 10.1111/j.1365-313X.2011.04722.x.

16. Weng H, Molina I, Shockey J, Browse J (2010) Organ fusion and defective cuticle function in a lacs1 lacs2 double mutant of Arabidopsis. Planta 231: 1089-1100.

17. Souza CD, Kim SS, Koch S, Kienow L, Schneider K, et al. (2009) A novel fatty acyl-CoA synthetase is required for pollen development and sporopollenin biosynthesis in Arabidopsis. Plant Cell 21: 507-525.
